# Construction of Novel Gene Signature-Based Predictive Model for the Diagnosis of Acute Myocardial Infarction by Combining Random Forest With Artificial Neural Network

**DOI:** 10.3389/fcvm.2022.876543

**Published:** 2022-05-25

**Authors:** Yanze Wu, Hui Chen, Lei Li, Liuping Zhang, Kai Dai, Tong Wen, Jingtian Peng, Xiaoping Peng, Zeqi Zheng, Ting Jiang, Wenjun Xiong

**Affiliations:** ^1^Department of Cardiology, The First Affiliated Hospital of Nanchang University, Nanchang, China; ^2^Jiangxi Medical College, Nanchang University, Nanchang, China; ^3^Department of Hospital Infection Control, The First Affiliated Hospital of Nanchang University, Nanchang, China

**Keywords:** acute myocardial infarction, predictive model, novel gene signatures, random forest, artificial neural network

## Abstract

**Background:**

Acute myocardial infarction (AMI) is one of the most common causes of mortality around the world. Early diagnosis of AMI contributes to improving prognosis. In our study, we aimed to construct a novel predictive model for the diagnosis of AMI using an artificial neural network (ANN), and we verified its diagnostic value *via* constructing the receiver operating characteristic (ROC).

**Methods:**

We downloaded three publicly available datasets (training sets GSE48060, GSE60993, and GSE66360) from Gene Expression Omnibus (GEO) database, and differentially expressed genes (DEGs) were identified between 87 AMI and 78 control samples. We applied the random forest (RF) and ANN algorithms to further identify novel gene signatures and construct a model to predict the possibility of AMI. Besides, the diagnostic value of our model was further validated in the validation sets GSE61144 (7 AMI patients and 10 controls), GSE34198 (49 AMI patients and 48 controls), and GSE97320 (3 AMI patients and 3 controls).

**Results:**

A total of 71 DEGs were identified, of which 68 were upregulated and 3 were downregulated. Firstly, 11 key genes in 71 DEGs were screened with RF classifier for the classification of AMI and control samples. Then, we calculated the weight of each key gene using ANN. Furthermore, the diagnostic model was constructed and named neuralAMI, with significant predictive power (area under the curve [AUC] = 0.980). Finally, our model was validated with the independent datasets GSE61144 (AUC = 0.900), GSE34198 (AUC = 0.882), and GSE97320 (AUC = 1.00).

**Conclusion:**

Machine learning was used to develop a reliable predictive model for the diagnosis of AMI. The results of our study provide potential gene biomarkers for early disease screening.

## Introduction

Acute myocardial infarction (AMI) remains the leading cause of death and disability around the world (1, 2). Despite improvements in the pharmacological approaches to improve patients with AMI, their prognosis remains generally poor. Besides, with the global population age and the increase in life expectancy, diagnosis and prevent of AMI are more imperative than ever. Early diagnosis and interventional treatment of AMI significantly contribute to improving the prognosis of AMI patients (3). Currently, the evaluation of cardiac biomarkers, such as cardiac troponin I and cardiac troponin T, is considered one of the gold-standard tools for the diagnosis of AMI. However, the diagnosis of AMI based on these biomarkers is still unsatisfactory due to the limitations in sensitivity and specificity (4, 5).

In recent years, microarray technology has been widely used and improved. Rapid development in the field of bioinformatics provides novel methods for the prediction of AMI. Various biomarkers were identified using integrated bioinformatics, such as MMP9, TLR2, and ILB1, and they can be regarded as predictive and diagnostic tools for AMI (6, 7). Moreover, it has been reported that a multi-biomarker approach may significantly enhance the diagnostic accuracy for AMI (8). However, because of the complex genetic architecture, they may lack powerful ability. In such cases, poor accuracy, low efficiency, and lack of early screening prompt us to establish a novel predictive model for AMI. Huang et al. constructed an optimal prediction model for AMI using multiple machine-learning algorithms (area under the curve [AUC] = 0.794) (4). Obviously, this predictive model is not efficient enough for screening and early detection of AMI. Fortunately, the development of machine-learning techniques, such as random forest (RF) and artificial neural network (ANN), has been successfully applied in biomarker discovery (9, 10).

**FIGURE 1:**
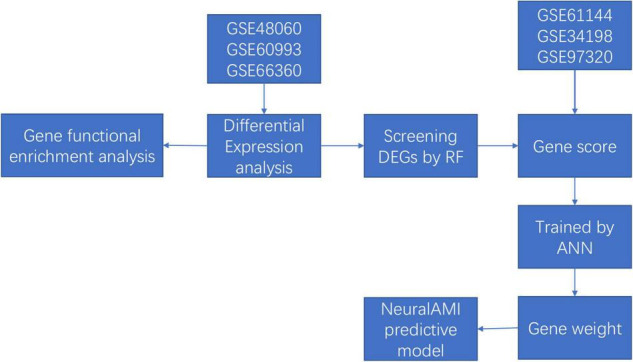
The flowchart of our study.

Thus, in our study, we combined the utilization of RF and ANN to construct a diagnostic model of AMI with microarray data from Gene Expression Omnibus (GEO) database. Firstly, we used the RF classifier to identify AMI-specific genes for classification, and the ANN algorithm was conducted to calculate the weights of each key gene. Then, we established a scoring model named neuralAMI with the cooperation of RF and ANN. Finally, we evaluated the classification performance of our model using the receiver operating characteristic (ROC) curve and validated this model with an independent dataset from GEO. The analysis process of our study is shown in Figure 1.

## Materials and Methods

### Gene Expression Profile Data Collection

In our research, we searched the datasets, which contained patients AMI and normal controls. The microarray expression datasets (GSE48060, GSE60993, GSE66360, GSE61144, GSE34198, and GSE97320) were downloaded from the GEO database^1^ (11–13). The gene expression profiling dataset GSE48060 contained 49 AMIs and 50 controls collected from blood, while GSE66360 included 31 AMIs and 21 controls collected from circulating endothelial cells, which were both using the GPL570 platform of Affymetrix Human Genome U133 Plus 2.0 Array. In addition, the GSE60993 dataset included 7 AMIs and 7 controls collected from peripheral blood, which is based on the GPL6884 platform of Illumina HumanWG-6 v3.0 Expression BeadChip. In addition, the GSE61144 dataset was used as the validation set, which collects 7 AMIs and 10 control samples based on the GPL6106 platform of Sentrix Human-6 v2 Expression BeadChip, the dataset GSE34198 collects 49 AMIs and 48 controls based on GPL6102 platform of Illumina Human-6 v2.0 Expression BeadChip, and GSE97320 collects 3 AMIs and 3 controls using the GPL570 platform of Affymetrix Human Genome U133 Plus 2.0 Array.

### Differentially Expressed Genes Identification

Preprocessing step was performed before differentially expressed gene (DEG) analysis. If a gene had multiple probe sites during the conversion of probe ID and gene symbol, we used the averaged values of the probe sites as a gene expression level. Besides, we converted the probe IDs to the genes symbol based on the annotation files from the respective platform and removed the probes that did not correspond to the genes symbol.

Moreover, the three datasets were merged into a training cohort, and the batch effects were preprocessed and removed by the ComBat function of the SVA package (14). DEGs between patients with AMI and controls were identified by utilizing the “limma” package of R software (15). Moreover, the “limma” package was also used to conduct background correction and data normalization between arrays. We set the threshold of DEGs in the dataset as adjusted value of *p* < 0.05 and |log_2_FC| > 1 (FC: fold change). Besides, we constructed a logistic regression model to verify the value of DEGs in segregating patients using SPSS22.0.

### Gene Functional Enrichment Analysis of Differentially Expressed Genes

The “clusterProfiler” and “GOplot” packages of R software were utilized to analyze the significant DEGs and to perform the Gene Ontology (GO) enrichment and Kyoto Encyclopedia of Genes and Genomes (KEGG) pathway enrichment analyses (16–18). False discovery rate (FDR) < 0.05 was considered as the significantly enriched gene sets.

Metascape^2^ was also used to conduct biological processes and pathway enrichment annotation, which provided a comprehensive idea for each gene (19). Terms with a value of *p* < 0.05 and a minimum count of 3 genes were considered significant. The significant terms were grouped into clusters based on their membership similarities, and the most statistically significant term within a cluster was chosen to represent the cluster.

### Screening Diagnostic-Related Differentially Expressed Genes With Random Forest

Random forest is a popular method for various prediction problems based on the classification tree (20). The randomForest package in R software was applied to construct an **RF** model for screening out the DEGs. Firstly, we calculated each error rate of 1–500 trees, and the optimal tree number was determined by the tree number with the lowest error rate and the best stability. Next, an RF was constructed based on the selected parameter, and the important genes were screened as the candidate genes for AMI diagnosis according to the decreasing accuracy method (Gini coefficient method). The gene importance greater than 2 is a common screening criterion in the RF algorithm, which has been used in similar studies (21). Finally, the top 11 DEGs were chosen as the novel gene signatures for the predictive model in AMI.

### Development of the Predictive Model With Neural Network

Firstly, we converted the expression data of the 12 DEGs into “Gene Score” based on their expression levels with the min-max method, which was conducted to normalize the data. Take one sample, for example, if the expression value of a downregulated gene in a certain sample is higher than the median expression value of the gene in all samples, its expression will be valued as 0, otherwise 1. Similarly, if the expression value of an upregulated gene is higher, its expression will be valued as 1, otherwise 0. Above all, the “Gene Score” sheet is composed of 165 lines of samples and 11 columns of DEGs.

The neuralnet package in R software (version 4.1.2) was used to establish an ANN model (22). The ANN contained one input layer, one hidden layer, and one output layer. The number of hidden layers was set as 3 and we set two nodes (control/AMI) in the output layer. Thus, we constructed a classification model of AMI disease with information on gene weight. After the ANN training, the calculation formula called neuralAMI was constructed, which could evaluate the classification score of the AMI disease. Besides, we used the pROC package in R software (version 4.1.2) to calculate the verification results of the predictive performance of our model constructed in our study.


NeuralAMI=Σ⁢(GeneExpression×NeuralNetworkWeight)

### Validation of the Predictive Model by Area Under the Curve

The datasets GSE61144, GSE34198, and GSE97320 were used to validate the effectiveness of the classification score model based on the training datasets. We screened out the DEGs in Gse61144, GSE34198, and GSE97320. In addition, the expression levels of DEGs were converted into binary status (above the median or below the median) according to the conversion methods mentioned above. Thus, an updated “Gene Score” was obtained. The neuralAMI of the validation set was calculated by the summation of “GeneExpression” × “NeuralNetworkWeight.” The AUC was calculated using the pROC package in R software (version 4.1.2), which was considered as an indicator to estimate the predictive capability of the ANN model.

## Results

### Identification of Differentially Expressed Genes in Acute Myocardial Infarction

**FIGURE 2:**
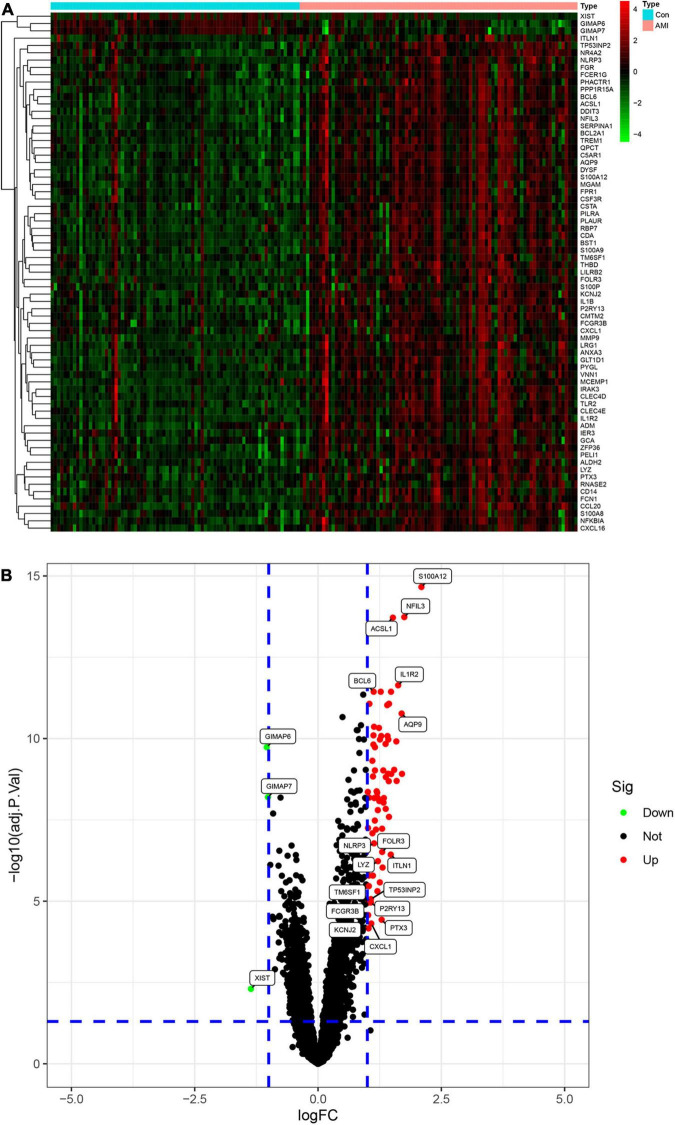
Analysis of differential expression genes (DEGs) in the training set. **(A)** The heatmap of all the DEGS in the training set. Red color means a higher expression level and green color means a lower expression level. **(B)** Volcano plot of differential expression of analysis results. In the map, each red spot represents an upregulated gene, while each green spot represents a downregulated gene.

**FIGURE 3:**
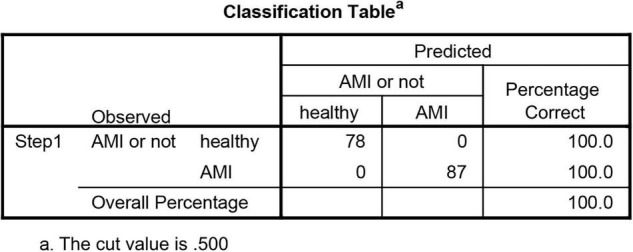
Logistic regression model of the value of differential expression genes (DEGs) in segregating patients.

We merged three datasets (GSE48060, GSE60993, and GSE66360) into a training cohort. After removing the batch effects, we used the “limma” package in R software to identify the DEGs. In total, 71 DEGs were identified that include 68 genes that were significantly upregulated and 3 genes that were significantly downregulated, as shown in Figure 2B, also known as the volcano plots. The DEG expression levels are presented in Figure 2A. These 71 DEGs are well performing in segregating patients (Figure 3).

### Functional Enrichment Analysis of Differentially Expressed Genes

**FIGURE 4:**
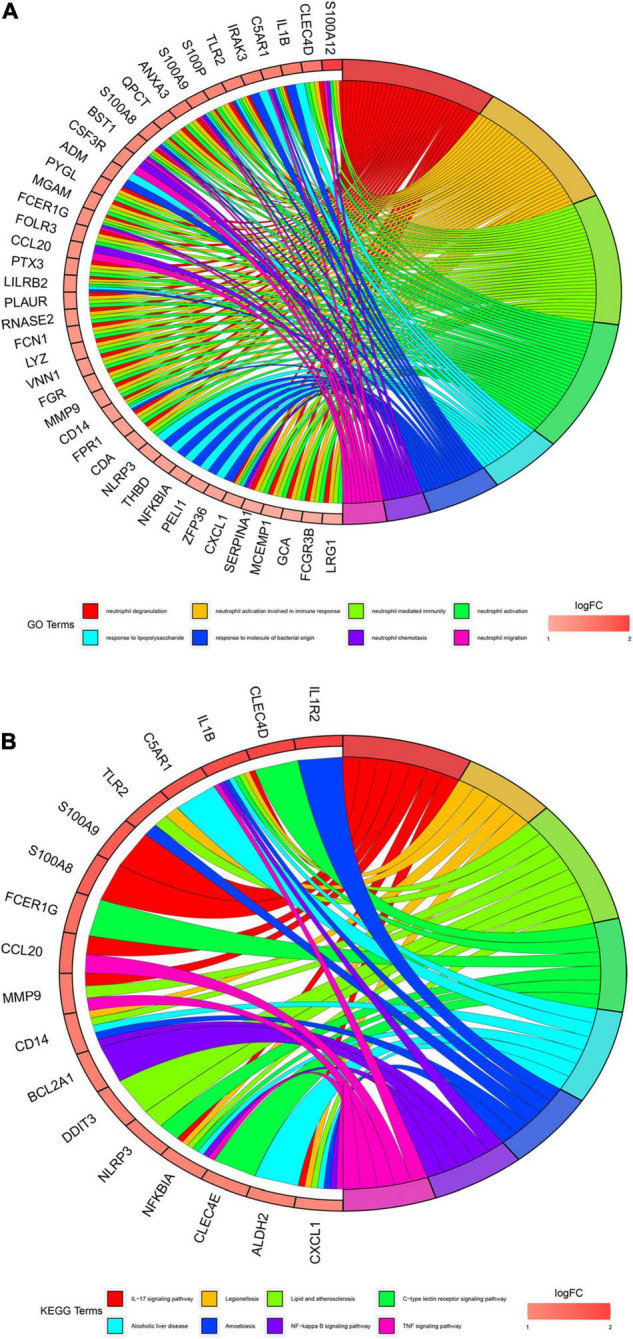
Gene enrichment analysis results. **(A)** Gene Oncology (GO) enrichment analysis of 71 differential expression genes (DEGs). The graph shows the relationships between DEGs and the top 8 enriched GO terms. Upregulated DEGs are in red color and downregulated DEGs are in blue color. **(B)**. Kyoto Encyclopedia of Genes and Genomes (KEGG) pathway enrichment analysis of 71 differential expression genes (DEGs). The graph shows the relationships between DEGs and the top 8 enriched KEGG pathways. Upregulated DEGs are in red color and downregulated DEGs are in blue color.

We conducted gene enrichment analysis to furthermore exploit the functions of DEGs. GO and KEGG analyses were performed on 71 DEGs. Results of GO analysis showed that DEGs were mainly enriched in immune-related biological processes, such as “neutrophil degranulation,” “neutrophil activation involved in immune response,” and “neutrophil-mediated immunity.” KEGG pathway analysis suggested that DEGs primarily include immune-related pathways, such as the “Interleukin (IL)-17 signaling pathway,” “C-type lectin receptor signaling pathway,” and “NF-kappa B signaling pathway” (Figure 4). These enrichments were upregulated in AMI, indicating their inhibitors can be regarded as potential therapeutic strategies for patients with AMI.

**FIGURE 5:**
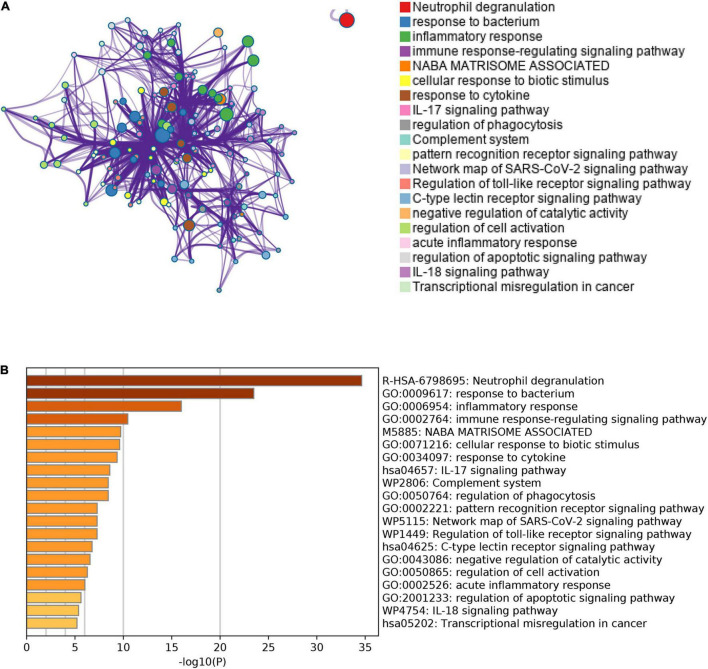
The results of Metascape analysis. **(A)** The network of enriched terms. The top 20 clusters were selected and rendered as a network, in which terms with a similarity score > 0.3 are connected by an edge. The thickness of the edge represents the similarity score. **(B)** Bar graph of enriched terms. The bar was colored by values of *p*. The lower the values of *p*, the deeper the color.

Besides, we also performed the enrichment analysis using Metascape. The Metascape analysis showed the top 20 clusters that DEGs were significantly enriched (Figure 5). The Metascape analysis showed results similar to Figure 4, which confirmed our results.

### Random Forest Screening for Candidate Acute Myocardial Infarction-Specific Differentially Expressed Genes

**FIGURE 6:**
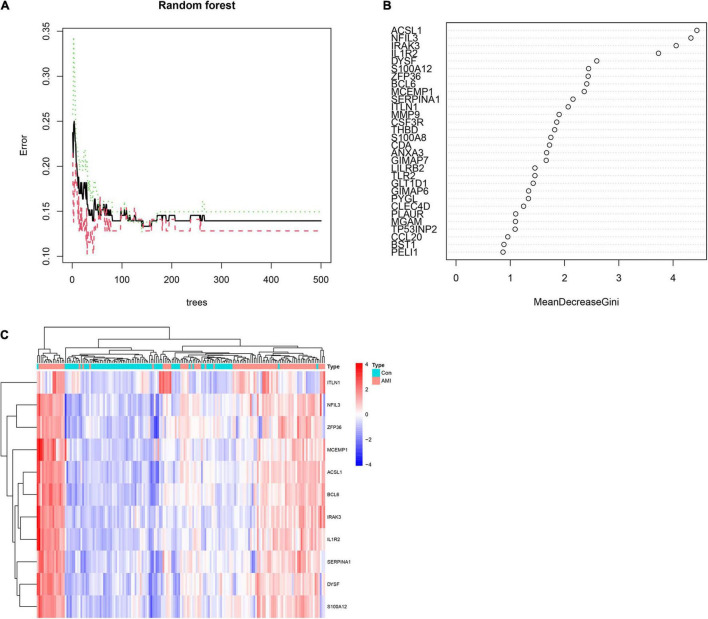
Identify the Acute myocardial infarction (AMI)-specific genes using random forest. **(A)** The influence of the number of decision trees on the error rate. The *x*-axis represents the number of decision trees and the *y*-axis is the error rate. **(B)** The top 30 differential expression genes (DEGs) of the Gini coefficient method are based on random forest classifier. The *x*-axis represents the importance index, and the *y*-axis represents the genes. **(C)** Heatmap of the top 11 key genes. Red color means genes with high expression, while blue color means genes with low expression.

To obtain AMI-specific genes, we applied the expression data of 71 DEGs to the RF classifier. Afterward, to adjust the parameters of the RF model, each error rate of 1–500 trees was calculated. As shown in Figure 6A, we choose the best ntree value (ntree = 140) due to the lowest error rates. The top 30 in the results of MeanDecreaseGini are presented in Figure 6B. Finally, we identified a set of 11 AMI-specific DEGs with an importance greater than 2 for subsequent analysis. The heatmap shows the expression level of the 11 AMI-specific DEGs, which are presented in Figure 6C. These 11 genes belong to a cluster with high expression in the AMI samples and low expression in the control samples.

### Artificial Neural Network-Based Establishment of the Acute Myocardial Infarction Predictive Model

**FIGURE 7:**
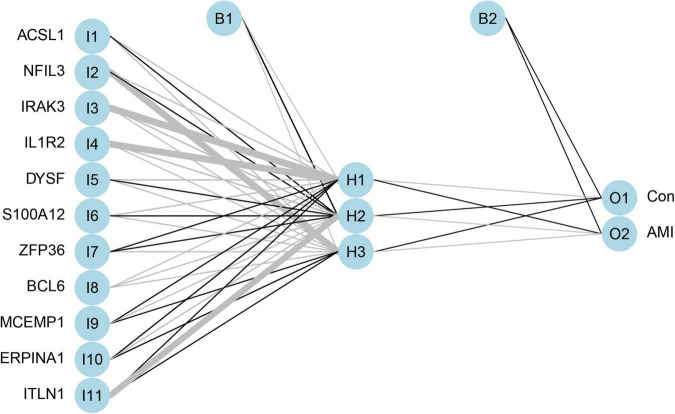
The visualization of artificial neural network. The neural network contains 11 input layers, 5 hidden layers, and 2 output layers.

**FIGURE 8:**
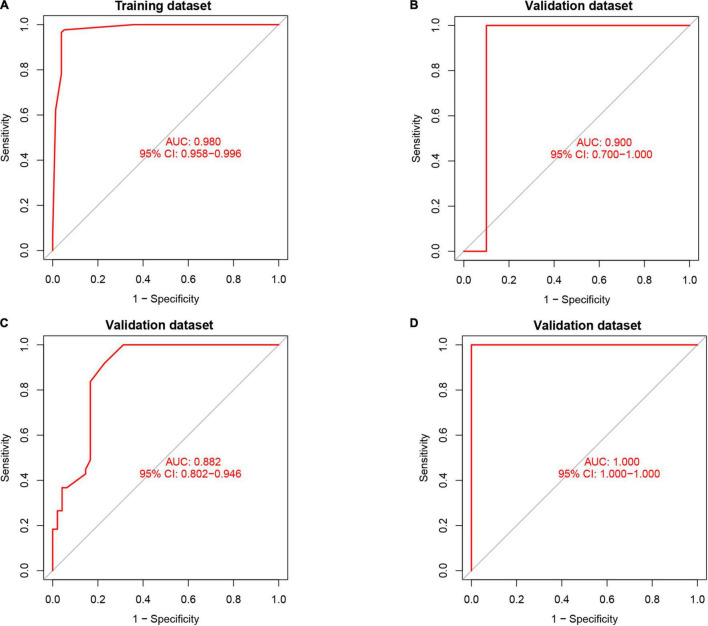
The results of the receiver operator curve (ROC) verification. **(A)** Training dataset. **(B)** Validation dataset (GSE61144). **(C)** Validation dataset (GSE34198). **(D)** Validation dataset (GSE97320).

Artificial neural network analysis was performed to optimize the weight of each gene based on the expression transformation of AMI-specific genes into “Gene Score.” ANN model includes 11 input layers, 3 hidden layers, and 2 output layers (Figure 7). The AMI-specific scoring model was calculated by the summation of “GeneExpression” × “NeuralNetworkWeight,” which can classify the gene expression levels between AMI and control samples. Besides, the detailed information on gene weight is shown in Supplementary Table 1. We can see that the entire training was conducted in 3,369 steps and the termination condition (reached threshold) was that the absolute partial derivative of the error function was <0.01. With the calculation of the pROC package (R version 4.1.2), the AUC for our predictive model was 0.980, indicating the model has outstanding discrimination (Figure 8A).

### Validation of NeuralAMI

To find out whether the neuralAMI model can predict the occurrence of AMI, three independent datasets GSE61144, GSE34198, and GSE97320 were introduced to validate the model we constructed. Similarly, we used an RF algorithm to identify the AMI-specific DEGs in the validation sets. Then the “Gene Score” and neuralAMI of GSE61144 were calculated in the same way as training sets. In addition, the AUC values of the validation model were 0.900, 0.882, and 1.00, which confirmed that our model exhibits high stability and validity (Figures 8B–D).

## Discussion

With the development of machine-learning algorithms and the availability of gene expression information in public databases, we have the opportunity to identify the biomarkers for the diagnosis or prognosis of the disease in various other fields (23, 24). In the field of AMI diagnosis, various attempts have been conducted to explore a diagnostic better method for AMI using diverse machine-learning algorithms (25, 26). In our study, we aimed to construct a diagnostic model for AMI based on the datasets from the GEO database. Firstly, we identified 11 AMI-specific DEGs *via* an RF classifier, and the ANN was used to calculate the weight of these genes. Then we developed the scoring model neuralAMI and constructed ROC to validate the accuracy and stability of the diagnostic model we established.

The enrichment analysis of DEGs indicates that most of the screened DEGs were mainly involved in the immune response. We found that neutrophil response-related biological processes. In practice, it has been reported that neutrophils are involved in the development of AMI, and we can improve AMI by suppressing the activation of neutrophil (27, 28). In addition, the “IL-17 signaling pathway” is the best-characterized pathway among the KEGG pathways. Recent studies have reported that IL-17 plays a role in the development of AMI (6, 29). Thus, the IL-17 signaling pathway may be a novel therapeutic target for AMI. There have also been reported that the IL-17 level in patients with AMI is markedly higher than in controls, which indicates that IL-17 might be a biomarker for AMI (30). The correct interpretation of DEG enrichment analysis results contributes to reveal the molecular mechanism of AMI and discover novel diagnostic predictors and therapeutic targets.

In this study, the top 11 key genes in DEGs were screened by the RF model based on MeanDecreaseGini. Furthermore, 10 of the 11 genes were also considered as AMI candidate genes in other studies: Acyl-CoA synthetase long-chain family member 1 (ACSL1) (31), nuclear factor interleukin-3-regulated (NFIL3) (32), interleukin-1 receptor-associated kinase 3 (IRAK3) (33), interleukin-1 receptor type 2 (IL1R2) (7), dysferlin (*DYSF)* (32), S100A12 (34), B-cell lymphoma 6 (BCL-6) (35), mast cell-expressed membrane protein 1 (*MCEMP1*) (36), serpin family A member 1 (*SERPINA1*) (37), and intelectin-1 (*ITLN1*) (38). Besides, we identified for the first time that zinc finger protein-36 (ZFP36) may get involve in the pathogenesis of AMI. It was reported that the expression of ZFP36 was low in the healthy aorta but was high in endothelial cells covered with atherosclerotic lesions in human (39).

The application of RF and ANN in constructing disease models has proven to be sophisticated (21). The highlighted novelty of our diagnostic model was firstly integrated RF and ANN algorithms to improve the predictive ability of the AMI predictive model. In addition, our scoring model was achieved by comprehensively considering the genes and their weight, which is vital to classify AMI and control samples. Gene weight is the actual value associated with each gene, indicating the importance of that gene in predicting the final output value. Besides, we removed the batch effects of training datasets and validation datasets by gene scoring, thus improving the predictive power of the model. As one of the most important machine-learning approaches, the advantages of RF include relatively good accuracy, precision, and ease of use, contributing to recognizing the key genes (40). The ANN algorithm has a good predictive ability, and the ANN model is stable and reliable (41, 42). It has been reported that the combined machine-learning methods of RF and ANN were efficient in many data-generating processes (43). As shown in our study, our model has a superior predictive capacity (AUC = 0.980) when compared to another model built by Chen et al. (AUC = 0.8550) (44). Moreover, the AUC of the predictive model achieved 0.900 in the validation set GSE61144, 0.882 in set GSE34198, and 1.00 in set GSE97320, which suggested that our model is of great applicability. The high AUC score in our predictive model indicates it could distinguish between AMI and control samples with a promising probability in microarray data.

However, there are still some limitations to our study. First, even though our predictive model is equipped with satisfactory performance in training and validation sets, the sample size in each dataset is relatively small. Thus, we combined 3 and 3 small-size datasets to get microarray training and validation sets. Second, the construction of the predictive model was based on the datasets from the GEO database, we should conduct *in vitro* and *in vivo* experiments to practice and verify our predictive model. Last but not least, because of the limitation of sample size, we did not conduct a fivefold cross-validation in ANN analyses. However, the expression data in our study are from peripheral blood and circulating endothelial cells, thus the predictive model can be applied to determine the likelihood of AMI through timely blood tests or tissue biopsy, which suggests our model has an outstanding classification performance. There is no doubt that our diagnostic model has a certain clinical value. Our predictive model needs to be investigated further in clinical work.

## Conclusion

We established a novel predictive model for AMI based on machine-learning algorithms and a neuralAMI scoring formula that could be used to predict patients with AMI. Besides, we validated the model with an independent dataset from GEO. Our study provides clinicians with a novel diagnostic strategy that shows better prediction performance than using existing biomarkers.

## Data Availability Statement

The datasets presented in this study can be found in online repositories. The names of the repository/repositories and accession number(s) can be found in the article/Supplementary Material.

## Author Contributions

YW and WX conceived and designed the study. HC, LL, LZ, KD, TW, JP, XP, ZZ, and TJ collected the data and assisted in data analysis. TJ, HC, LL, and WX provided the significant suggestions on the methodology. YW conducted the data management and bioinformatics analysis. YW and HC drafted the manuscript. LL, LZ, KD, TW, JP, XP, ZZ, and TJ edited and revised the article. All authors read and approved the final manuscript.

## Conflict of Interest

The authors declare that the research was conducted in the absence of any commercial or financial relationships that could be construed as a potential conflict of interest.

## Publisher’s Note

All claims expressed in this article are solely those of the authors and do not necessarily represent those of their affiliated organizations, or those of the publisher, the editors and the reviewers. Any product that may be evaluated in this article, or claim that may be made by its manufacturer, is not guaranteed or endorsed by the publisher.

## Footnotes

1
http://www.ncbi.nlm.nih.gov/geo/

2
http://metascape.org
